# Developmental Origins of Physical Fitness: The Helsinki Birth Cohort Study

**DOI:** 10.1371/journal.pone.0022302

**Published:** 2011-07-22

**Authors:** Minna K. Salonen, Eero Kajantie, Clive Osmond, Tom Forsén, Hilkka Ylihärsilä, Maria Paile-Hyvärinen, D. J. P. Barker, Johan G. Eriksson

**Affiliations:** 1 Department of Chronic Disease Prevention, National Institute for Health and Welfare, Helsinki, Finland; 2 Department of Public Health, University of Helsinki, Finland; 3 Epidemiology Resource Centre, Southampton, United Kingdom; 4 Vaasa Health Care Centre, Diabetes Unit, Vaasa, Finland; 5 Developmental Origins of Health and Disease Division, University of Southampton, Southampton, United Kingdom; 6 Department of General Practice and Primary Health Care, University of Helsinki, Helsinki, Finland; 7 Helsinki University Central Hospital, Unit of General Practice, Finland; 8 Vaasa Central Hospital, Vaasa, Finland; 9 Folkhälsan Research Center, Helsinki, Finland; Universidad Peruana Cayetano Heredia, Peru

## Abstract

**Background:**

Cardiorespiratory fitness (CRF) is a major factor influencing health and disease outcomes including all-cause mortality and cardiovascular disease. Importantly CRF is also modifiable and could therefore have a major public health impact. Early life exposures play a major role in chronic disease development. Our aim was to explore the potential prenatal and childhood origins of CRF in later life.

**Methods/Principal Findings:**

This sub-study of the HBCS (Helsinki Birth Cohort Study) includes 606 men and women who underwent a thorough clinical examination and participated in the UKK 2-km walk test, which has been validated against a maximal exercise stress test as a measure of CRF in population studies. Data on body size at birth and growth during infancy and childhood were obtained from hospital, child welfare and school health records. Body size at birth was not associated with adult CRF. A 1 cm increase in height at 2 and 7 years was associated with 0.21 ml/kg/min (95% CI 0.02 to 0.40) and 0.16 ml/kg/min (95% CI 0.03 to 0.28) higher VO_2max_, respectively. Adjustment for adult lean body mass strengthened these findings. Weight at 2 and 7 years and height at 11 years became positively associated with CRF after adult lean body mass adjustment. However, a 1 kg/m^2^ higher BMI at 11 years was associated with −0.57 ml/kg/min (95% CI −0.91 to −0.24) lower adult VO_2max_, and remained so after adjustment for adult lean body mass.

**Conclusion/Significance:**

We did not observe any significant associations between body size at birth and CRF in later life. However, childhood growth was associated with CRF in adulthood. These findings suggest, importantly from a public point of view, that early growth may play a role in predicting adult CRF.

## Introduction

Cardiorespiratory fitness (CRF) is a good indicator of overall health. Physical activity and CRF are associated with the risk of all cause mortality, cardiovascular disease and several cancers [1]–[6]. It has been also suggested, that CRF is associated with brain structure and function [7] and that improvements in CRF brought about by exercise training are implicated in the restoration of neural and cognitive functioning in older adults [8]–[10]. Animal models and epidemiological studies in humans propose that physical activity and CRF may have a prenatal origin [11]–[16].

Babies who grow at slow rates in utero and thus end up being small or thin at birth, are at increased risk of developing cardiovascular disease (CVD), type 2 diabetes and components of the metabolic syndrome [17]–[21] in later life. Major influences of postnatal growth patterns on later health are also well established. While results of several studies suggest that slow gain in body size during the first two years of life is associated with cardiovascular disease and its risk factors [22]–[24], including type 2 diabetes [23] and dyslipidemia [25], [26], the others have shown the opposite effect of growth during infancy [27], [28] on these diseases and the risk factors. Postnatal conditions including dietary and exercise habits and other behavioral related aspects are mostly modifiable and hence important from a public health point of view.

Recent findings from the NordNet Study showed that the associations found between birth weight and leisure time physical activity (LTPA) are weak within the normal birth weight range. However, both low and high birth weights were associated with lower LTPA [12]. In a previous Finnish study, a low birth weight was associated with the metabolic syndrome, but not among those who were fit and active [13]. Our previous findings in a cohort of men and women born 1924–33 suggest that people born thin or with a low birth weight benefit most from physical activity and exercise in adult life by getting the biggest protective effect against diabetes [14]. More recent findings from Helsinki Birth Cohort Study on people born 1934–44 propose that those who were taller and heavier as children, tend to engage more in leisure time physical activity in adult life compared to their shorter and lighter peers [29].

While physical activity and CRF are tightly related to each other, associations of CRF with many health outcomes remain when physical activity levels are adjusted for. In addition, associations with health tend to be stronger for CRF than for physical activity [30]. However, whether CRF in adult life is associated with early growth has been little studied. Most previous publications focus on children, adolescents or young adults [15], [16], [31]. Our aim was to examine whether early growth during childhood, from birth to 11 years predicts CRF in later life. We hypothesized that small body size at birth, and slow growth during infancy and childhood would be associated with lower CRF levels in late middle aged men and women.

## Methods

This study is part of the Helsinki Birth Cohort Study (HBCS) which in the epidemiological part included 8760 men and women born at Helsinki University Central Hospital. They were born between 1934 and 1944, attended child welfare clinics in the city and were living in Finland in 1971, when a unique identification number was assigned to all residents of Finland. The majority of the children (77%) also went to school in Helsinki. The birth records included data on weight and length at birth and gestational age. Records from child welfare clinics and school health care include serial measurements of weight and height. On average, the participants had 11 measurements between birth and two years, and 9 measurements between 2 and 11 years. Details of the birth, child welfare and school health records have been described in detail elsewhere [32].

Of the original cohort, 2003 randomly selected individuals participated in a detailed clinical examination between the years 2001–2004. The clinical study protocol was approved by the Ethics Committee of Epidemiology and Public Health of the Hospital District of Helsinki and Uusimaa. Written informed consent was obtained from each participant before any study procedure was initiated.

All measurements were taken by a team of trained research nurses. Height was measured in light indoor clothing without shoes to the nearest 0.1 cm and weight to the nearest 0.1 kg. Body mass index (BMI) was calculated as weight in kilograms divided by the square of height in meters. Waist circumference was measured midway between the lowest rib and the iliac crest. Lean body mass and body fat mass were measured by bioelectrical impedance analysis (BIA) using InBody 3.0 eight-polar tactile electrode system (Biospace Co. Ltd, Seoul, Korea) [33].

Using the occupations recorded on the birth, child welfare clinic and school health care records, we grouped fathers into three socioeconomic groups (manual workers, lower-middle and upper-middle classes), originally based on a nine-group social classification system by Statistics Finland. We used the highest group obtained from these three sets of records. Social class in adulthood, based on subjects' own occupation, was derived from Census data in 1980. Physical activity and smoking habits were obtained from a self-administered questionnaire. Being physically active was defined as taking at least moderate exercise three or more times per week. Subjects were defined as smokers if they smoked one or more cigarettes per day.

In the present study, we restricted our analyses to the 606 individuals who took part in the UKK 2-kilometer walk test, in order to examine the effects of fetal and childhood growth on cardiovascular performance among normally active men and women in later life. The UKK 2-km walk test is an indirect measurement of CRF, which can be used within a majority of the adult population without requirements of maximal physical efforts. It has been validated against maximal effort test by treadmill or bicycle ergometry in multiple populations including the obese and elderly [34], [35] During the test, subjects are directed to walk through a 2-km course on the flat ground as rapidly as possible. The test results are expressed as fitness index, or alternatively as VO_2max_, by using a formula in which subject's age, sex, BMI, time spent in walking and heart rate are taken in to account.

Several health status requirements for the UKK walk test were set. A subject was excluded if he/she had any of the following conditions affecting the walk test: myocardial infarction within the past year, unstable blood pressure, CHD with symptoms, active arthritis or other joint disorder with pain, arrhythmia, asthma or other breathing difficulties, and if the subject had other medical restrictions concerning physical activity. Subjects on drug therapy affecting pulse rate were also excluded from the analyses. Individuals (n = 25) whose test result indicated a VO_2max_ status under 10 ml/kg/min were excluded from further analysis because those with the VO_2max_ value of this low can not reach the intensity required in the UKK- test, and thus 581 individuals remained in the study.

### Statistical analysis

A multiple linear regression was used to assess the associations of birth size, childhood height, weight and BMI with CRF. The basic models were adjusted for age and in the pooled analysis also for sex. Further analysis included the adjustments for gestational age, maternal BMI, socioeconomic factors (social class in childhood and adult life), exercise habits, smoking status, and current lean body mass. The regression models were also investigated using a quadratic term for each of the childhood body size variables (height and weight at birth, 2, 7 and 11 years); however, there was no evidence for nonlinear associations.

We calculated z-scores (SD-score) for height, weight and BMI for each child at birth and at each birthday until 12 years of age. The z-score is the number of standard deviations by which an observation differs from the mean for the whole study group. As the children were not measured exactly on their birthdays, we obtained the z-score for birthdays by interpolating the available measurements (if a measurement had been recorded within two years of the particular age) and used these estimated values. Most of the visits to child welfare clinics occurred before age two years; fewer measurements were made between ages two years and enrolment at school. We examined the effects of gains in height, weight and BMI after birth during three periods of growth (0–2, 2–7 and 7–11 years) on CRF. In these analyses, we used the residual from linear regression where current height, weight or BMI was regressed on previous corresponding measurements. By this construction, the residuals, which we refer to as “conditional growth” are uncorrelated with the earlier size measurements and enable the effects of changes in height, weight and BMI during different growth periods to be distinguished [36], [37].

The adult clinical as well as childhood growth characteristics are presented separately for men and women. Interaction terms were created to explore potential interactions between gender and growth measures in association to CRF. Since the effect of growth on CRF was similar in both, boys and girls, pooled analyses are presented.

## Results

### Comparison between the UKK 2-km walk test participants and non-participants

Childhood growth and adult characteristics are presented in the table 1. From the original cohort of 2003 individuals, those 606 who took part in the UKK 2-km walk test showed no difference in adult social class, exercise habits, birth size or growth during childhood, except for birth length in women, which was slightly higher for those who did attend in the walk test (p = 0.04). In adulthood the participants had a smaller BMI, smaller waist circumferences and lower body fat percentages than those who did not participate (p<0.001 for all). The results in relation to anthropometric characteristics were similar for both sexes. Men who did not attend in the walk test, were more often smokers compared to those who did attend (p = 0.02).

**Table 1:** Adult clinical and childhood growth characteristics of the 581 UKK 2-km walk test participants.

	Men		Women	
	(n = 269)		(n = 312)	
ADULT LIFE	Mean	(SD)	Mean	(SD)
Age (years)	61.3	(2.5)	61.76	(3.1)
Weight (kg)	83.0	(11.3)	70.4	(10.6)
Height (cm)	177.2	(6.3)	163.5	(5.4)
BMI (kg/m^2^)	26.4	(3.1)	26.4	(3.9)
Waist circumference (cm)	96.9	(8.9)	87.0	(10.1)
Lean body mass (kg)	64.7	(7.7)	47.2	(5.1)
Percent body fat (%)	21.6	(4.8)	32.1	(6.1)
Social class in adulthood				
Higher official (%)	44		27	
Lower official (%)	28		60	
Self employed (%)	5		3	
Manual worker (%)	22		10	
Smoker				
(%)	20		16	
Exercise				
(%)	50		47	
VO_2max_ (ml/kg/min)	30.5	(8.2)	24.9	(5.9)
CHILDHOOD				
Birth weight (g)	3491	(499)	3393	(444)
Birth length (cm)	50.8	(2.1)	50.2	(1.8)
Ponderal index (kg/m^3^)	26.6	(2.0)	26.7	(2.2)
BMI (kg/m^2^)				
at birth	13.5	(1.2)	13.4	(1.2)
at 2 y	16.7	(1.1)	16.5	(1.2)
at 7 y	15.5	(1.2)	15.4	(1.3)
at 11 y	16.8	(1.5)	17.0	(1.9)
Social class in childhood				
Upper middle (%)	21		15	
Lower middle (%)	25		25	
Labourer (%)	54		60	

BMI = body mass index, mean value (s.d.) unless otherwise stated, smoker = smoking one or more cigarettes per day, exercise = proportion of people who exercised 3 or more times per week.

A comparison of the associations of childhood body size measurements on potential components of adult CRF did not show differences between the participants and non-participants. Effect of childhood body size on adult BMI, body fat percentage, lean body mass and grip strength were similar in both groups.

### Associations between adult anthropometric, socioeconomic and life style factors and CRF

Age in men (p = 0.01) and women (p<0.001), weight, BMI, waist circumference, body fat percentage and fat mass (p<0.001 for all) and lean body mass (p = 0.02 for men and p<0.001 for women) were all inversely associated with CRF. Adult social class in both men and women (p = 0.002 and p = 0.02) was also inversely associated with CRF. Mean VO_2max_ in men was 32.5 ml/kg/min in the highest and 28.6 ml/kg/min in the lowest social class, and correspondingly 26.2 ml/kg/min in the highest and 23.4 ml/kg/min in the lowest social class in women. Again, height was positively associated with CRF in men (p = 0.005) and in women (p<0.001).

**Figure 1:**
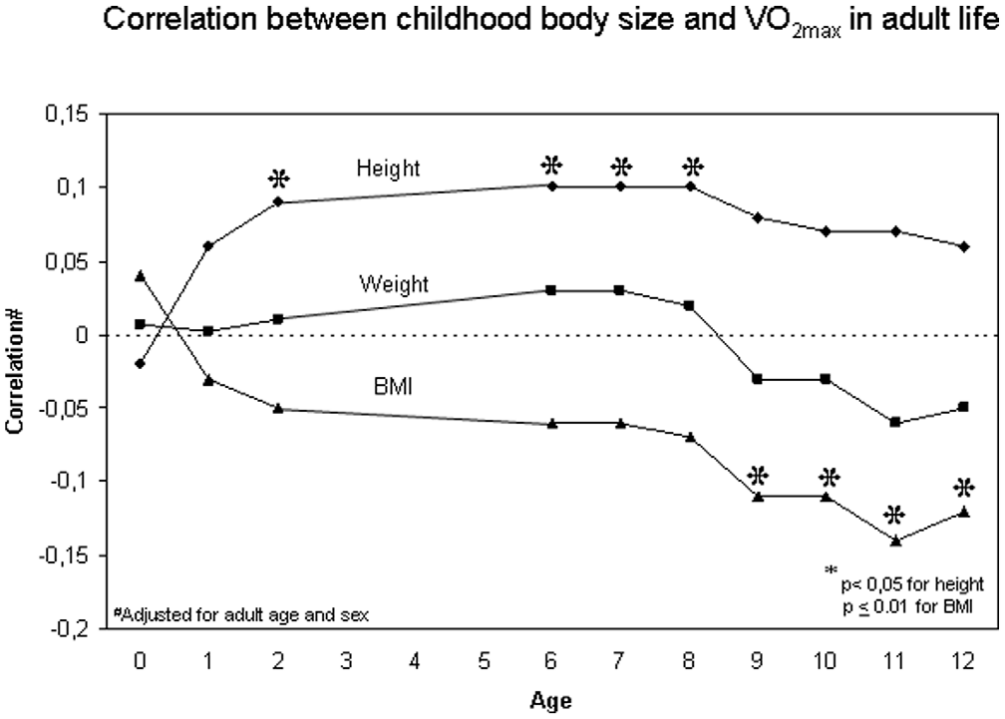
Age- and sex-adjusted correlations between VO2max and height, weight and BMI annually from birth to 12 years. The horizontal dashed line indicates a correlation of 0.

Men who took at least moderate exercise three or more times per week had higher CRF (mean VO_2max_ being 32.15 ml/kg/min compared to the 28.15 ml/kg/min in the inactive group, p = 0.001). This finding was not observed in women. A positive smoking status was associated with lower CRF in both men (p = 0.008) and in women (p = 0.005). Age and sex adjusted correlations between childhood body size and adult VO_2max_ are presented in figure 1.

### Impact of prenatal and childhood body size and socioeconomic factors on adult CRF

In the age and sex adjusted models birth length (table 2), birth weight (table 3), ponderal index, body mass index (table 4), gestational age and maternal BMI were not associated with CRF (VO_2max_) in adult life.

**Table 2:** Adult cardiorespiratory fitness in relation to length at birth and height at 2, 7, 11 years of age.

*VO_2max_	N	Mean (SD)	Mean (SD)	Mean (SD)	Mean (SD)
ml/kg/min		at birth (cm)	at 2 y (cm)	at 7 y (cm)	at 11 y (cm)
(Range)					
10.4–24.3	193	50.5 (2.1)	85.9 (3.0)	119.9 (4.9)	141.0 (6.5)
24.3–30.2	194	50.2 (1.9)	86.0 (2.9)	120.4 (4.6)	141.9 (6.2)
30.2–57.8	194	50.7 (1.8)	86.6 (3.1)	121.2 (4.6)	142.2 (5.6)
RC and		−0.06	0.21	0.16	0.08
95% CI		(−0.36, 0.24)	(0.02, 0.40)	(0.03, 0.28)	(−0.02, 1.17)
*p* for trend		*0.7*	*0.03*	*0.01*	*0.1*
1RC and		0.08	0.50	0.40	0.25
95% CI		−0.22, 0.39	(0.29, 0.71)	(0.26, 0.55)	(0.14, 0.36)
1 *p* for trend		*0.6*	*<0.001*	*<0.001*	*<0.001*
2RC and		0.10	0.41	0.34	0.21
95% CI		−0.20, 0.39	0.21, 0.61	0.21, 0.49	0.10, 0.31
2 *p* for trend		*0.5*	*<0.001*	*<0.001*	*<0.001*

*VO_2max_ in tertiles; RC = regression coefficients with 95% CI ( = confidence interval) express changes in VO_2max_ (ml/kg/min) per 1 cm increase in heights at birth, 2, 7 and 11 years; all the analysis were adjusted for age and sex;

1 adjusted additionally for adult lean body mass;

2 adjusted for age, sex, adult lean body mass, adult social class, exercise and smoking habits.

**Table 3:** Adult cardiorespiratory fitness in relation to weight at birth, 2, 7 and 11 years of age.

*VO_2max_	N	Mean (SD)	Mean (SD)	Mean (SD)	Mean (SD)
ml/kg/min		at birth (g)	at 2 y (kg)	at 7 y (kg)	at 11 y (kg)
(Range)					
10.4–24.3	193	3459 (513)	12.1 (1.2)	22.2 (2.7)	34.3 (5.6)
24.3–30.2	194	3361 (435)	12.1 (1.2)	22.4 (2.8)	34.1 (5.1)
30.2–57.8	194	3494 (458)	12.3 (1.1)	22.6 (2.8)	33.7 (4.8)
RC and		0.10	0.08	0.08	−0.08
95% CI		(−1.10, 1.30)	(−0.43, 0.58)	(−0.13, 0.30)	(−0.19, 0.03)
*p* for trend		*0.9*	*0.8*	*0.4*	*0.2*
1RC and		0.67	0.71	0.39	0.03
95% CI		(−0.56, 1.90)	(0.17, 1.26)	(0.15, 0.63)	(−0.09, 0.16)
1 *p* for trend		*0.3*	*0.01*	*0.001*	*0.6*
2RC and		0.79	0.75	0.36	0.001
95% CI		−0.41, 1.99	0.21, 1.30	0.13, 0.60	−0.12, 0.12
2 *p* for trend		*0.2*	*0.01*	*0.003*	*1.0*

*VO_2max_ in tertiles; RC = regression coefficient with 95% CI ( = confidence interval) express changes in VO_2max_ ml/kg/kg per1 kg increase in weight at birth, 2, 7 and 11 years; all the analysis were adjusted for age and sex;

1 adjusted additionally for adult lean body mass;

2 adjusted for age, sex, adult lean body mass, adult social class, exercise and smoking habits.

**Table 4:** Adult cardiorespiratory fitness in relation to BMI at birth, 2, 7 and 11 years of age.

*VO_2max_	N	Birth (kg/m^2^)	2 y (kg/m^2^)	7 y (kg/m^2^)	11 y (kg/m^2^)
ml/kg/min					
(Range)					
10.4–24.3	193	13.5 (1.3)	16.6 (1.2)	15.5 (1.2)	17.2 (1.8)
24.3–30.2	194	13.3 (1.1)	16.5 (1.1)	15.5 (1.3)	16.9 (1.7)
30.2–57.8	194	13.5 (1.1)	16.6 (1.2)	15.4 (1.2)	16.6. (1.6)
RC and		0.21	−0.30	−0.37	−0.57
95% CI		(−0.27, 0.69)	(−0.78, 0.18)	(−0.84, 0.11)	(−0.91, −0.24)
*p* for trend		*0.4*	*0.2*	*0.1*	*0.001*
1RC and		0.33	−0.06	−0.13	−0.43
95% CI		(−0.15, 0.81)	(−0.54, 0.43)	(−0.63, 0.36)	(−0.78, −0.08)
1 *p* for trend		*0.2*	*0.8*	*0.6*	*0.001*
2RC and		0.41	0.06	−0.05	−0.43
95% CI		−0.06, 0.87	−0.42, 0.53	−0.54, 0.43	−0.77, −0.10
2 *p* for trend		*0.09*	*0.8*	*0.8*	*0.01*

*VO_2max_ in tertiles; RC = regression coefficient with 95% CI ( = confidence interval) express changes in VO_2max_ (ml/kg/min) per 1 unit increase in BMI at birth, 2, 7 and 11 years; all the analysis were adjusted for age and sex;

1 adjusted additionally for adult lean body mass;

2 adjusted for age, sex, adult lean body mass, adult social class, exercise and smoking habits.

Height at 2 and 7 years were positively associated with CRF in the age and gender adjusted models (table 2). Adjustment for adult lean body mass strengthened these findings and height at 11 years became associated with CRF. Additional adjustments for childhood and adult social class, exercise and smoking habits did not change these findings.

Weight at 2 and 7 years of age became positively associated with CRF after adjustment for lean body mass (table 3) and the other confounders, such as social circumstances, exercise and smoking. Higher BMI at 11 years, independently of the confounders, predicted lower adult CRF (table 4).

Lower childhood social class was negatively associated with adult CRF (p = 0.04), especially in men (p = 0.003). Mean VO_2max_ was 33.3 ml/kg/min in the highest social class, 30.8 ml/kg/min in the second, and 29.3 ml/kg/min in the lowest group.

### Gains in body size from birth to 11 years and adult CRF

Associations between childhood growth and adult CRF are presented in the table 5.

**Table 5:** Association between childhood growth during the first 11years of life and adult cardiorespiratory fitness (VO_2max_).

		Model 1		Model 2
	β	95% CI	β	95% CI
Height (SD)				
0–2 y	0.72	0.13, 1.32	1.55	0.91, 2.19
2–7 y	0.40	−0.21, 1.00	0.97	0.35, 1.59
7–11 y	−0.27	−0.87, 0.32	−0.11	−0.70, 0.47
Weight (SD)				
0–2 y	0.02	−0.57, 0.61	0.69	0.05, 1.33
2–7 y	0.22	−0.39, 0.83	0.59	−0.04, 1.22
7–11 y	−1.10	−1.69, −0.51	−0.95	−1.54, −0.37
BMI (SD)				
0–2 y	−0.44	−1.03, 0.16	−0.22	−0.82, 0.38
2–7 y	−0.33	−0.93, 0.27	−0.23	−0.85, 0.38
7–11 y	−0.99	−1.58, −0.40	−0.87	−1.46, −0.28

β = regression coefficient; CI = confidence interval Height 7–11 y is the standardised residual of height at age 11 years regressed on heights at ages 0, 2, and 7 years. The conditional measures are adjusted on all earlier values.

The table describes the results of two regression analyses – for height and weight. All predictors are statistically uncorrelated by construction.

1) Model 1, measurement is adjusted for age and sex.

2) Model 2, measurement is adjusted for age, sex and current lean body mass.

In the age and sex adjusted models gain in height during infancy predicted higher adult CRF while gain in weight and BMI between 7 and 11 years was associated with lower adult CRF. However, when adult lean body mass was additionally adjusted for, gains in height between birth and 2 years remained and between 2 and 7 years became associated with higher CRF. Again, after additional adjustment for adult lean body mass, gain in weight during infancy became positively associated with adult CRF while gain in weight and BMI between 7 and 11 years remained negatively associated with adult CRF.

## Discussion

We hypothesized that non-optimal prenatal and childhood growth characterized by low birth weight and slow growth during childhood, predicts lower adult cardiorespiratory fitness (CRF). We did not find any significant associations between body size at birth and CRF in later life. However, childhood growth was associated with CRF in adulthood. We observed that children, who were taller at ages 2, 7 and 11 years, had higher CRF in their adult life, whereas higher BMI at 11 years of age predicted lower adult CRF. The positive association between childhood weight and adult CRF was largely explained by adult lean body mass.

An individual's CRF refers to ability of the body to supply oxygen to skeletal muscles during sustained physical activity. CRF is a physiological characteristic, commonly measured by a maximal or sub-maximal exercise test, whereas physical activity is a behavioral component, defined as any body movement that increases energy expenditure. Obviously, regular physical activity improves the cardiorespiratory system by increasing the amount of oxygen that is inhaled and distributed to body tissues and is thus a major determinant of CRF. As a behavioral component it is also a key target of interventions to reduce the risk of disease [38]–[40]. Both are important protective and modifiable factors when considering chronic diseases like type 2 diabetes, coronary heart disease and osteoporosis. In most studies associations with health outcomes tend to be stronger for CRF than for physical activity, although this may be in part explained by less accurate measurement of physical activity by self-report methods [30]. However, it has been proposed that CRF and physical activity may be differentially influenced by age, gender, social, environmental and behavioral factors as well as genotypes and subclinical disease [41]–[44].

Animal models have suggested that life style and exercise habits may have a prenatal origin. Severe malnourishment of a pregnant rat leads to a smaller body size at birth and inactivity in the offspring. Sedentary behaviour is further exacerbated by postnatal hypercaloric nutrition [11]. Human studies, however, suggest that the associations between prenatal characteristics and physical activity may be limited to extreme groups. For example, an extensive NordNet Collaboration study showed that the association between birth weight and leisure time physical activity was weak within the normal birth weight range, although both very low and high birth weights were associated with lower leisure time physical activity [12]. Accordingly, a recent study showed much reduced levels of physical activity in young adults born preterm with birth weight below 1500 g [45]. In the present study we found no evidence for a u-shaped relationship between birth measurements and CRF. This may be because when the study subjects were born, infants with very low birth weights were less likely to survive, and the rates of gestational diabetes, a key cause of high birth weight, are likely to have been lower than today. Our findings suggest that CRF in late adult life is affected by factors that affect growth during childhood. This is consistent with our recent findings on leisure time physical activity, assessed by a detailed questionnaire in the same cohort [29].

Mechanisms behind the relationship between early growth and CRF are still poorly understood. Muscle fitness may play a role: we have previously shown that low birth weight is associated with lower adult lean mass whereas grip strength, an important predictor of frailty and functional capacity in elderly people, was positively associated with birth weight through its association with lean mass [46]. Ortega and co-workers have shown that those who had higher birth weight also had higher hand grip strength from ages 13 to 18.5 years. However, the associations were mostly explained by fat-free mass and birth weight was not associated with CRF [16]. A study of Lawlor's and co-workers showed that those born bigger had higher CRF- levels at the age of 9 years [15]. Motor development may also play a role: The Northern Finland Birth Cohort showed that higher birth weight, lower infant weight gain and earlier infant motor development predict higher levels of adult physical performance for muscle strength, muscle endurance and aerobic fitness at the age of 31 years [31].

### Strengths and limitations of the study

We have unique data on both men and women with details on reliable birth and growth measurements and with a long follow up period. Body composition was measured with a validated BIA method [47] and CRF with the validated UKK 2-km walk test [34], [48], [49]. In addition to the present study, there are only a few studies examining strictly the relationship between early growth and CRF.

Limitations of our study have been discussed elsewhere [22], [32]. Our study was restricted to the men and women who were born in the Helsinki University Central Hospital, attended child welfare clinics which was voluntary, were still alive and living in Finland in the year 2003 and willing to participate to the clinical examination. They may not be representative of the population born in Helsinki. However, our analyses are based on internal comparisons within the cohort. Of the 2003 clinical participants 606 men and women attended the UKK 2-km walk test, which means that the power is lower than that in the original study. Despite the fairly large set of exclusion criteria, the childhood growth of those who participated in the walk test did not differ from those who did not. In addition to the childhood gains in growth, it would be valuable to evaluate other factors such as physical activity and dietary patterns during childhood, and their impact on adult body composition and CRF. Further more, early versus late puberty may well be part of the explanation for the mechanisms which play a role in the associations between childhood growth and adult CRF. Unfortunately such data are not available for our cohort.

### Conclusion

Besides the long term effect of birth size on adult body composition and several diseases that has been demonstrated in a large number of studies [17]–[19], [46], [50] growth during infancy and in childhood has been shown to play important roles in the pathogenesis of several diseases [22]–[27], [51], [52]. However, previous studies provide a good basis for the present study. The results of the present study emphasize a role of childhood height from the age of two years onwards and in particular the gain in height during infancy on CRF-level later in life. However, childhood height is strongly and positively correlated with adult stature, which is positively associated with CRF-level, and therefore it is difficult to disentangle the two. The observed associations between childhood height and CRF were independent of confounders including childhood and adult socio-economic circumstances, adult physical activity, smoking habits and adult lean body mass.
